# Cultural Impact on the Intention to Use Nursing Information Systems of Nurses in Taiwan and China: Survey and Analysis

**DOI:** 10.2196/18078

**Published:** 2020-08-12

**Authors:** I-Chiu Chang, Po-Jin Lin, Ting-Hung Chen, Chia-Hui Chang

**Affiliations:** 1 Department of Information Management National Chung Cheng University Chia-Yi Taiwan; 2 Institute of Health Policy and Management National Taiwan University Taipei Taiwan; 3 Department of Nursing Taichung Veterans General Hospital Taichung Taiwan; 4 Department of Nursing Hungkuang University Taichung Taiwan

**Keywords:** Nursing information system, intention to use, cultural differences, information literacy

## Abstract

**Background:**

Nursing workforce shortage has emerged as a global problem. Foreign nurse importation is a popular strategy to address the shortage. The interactions between nursing staff on either side of the Taiwan Strait continue to increase. Since both nurses in Taiwan and nurses in China have adopted nursing information systems to improve health care processes and quality, it is necessary to investigate factors influencing nursing information system usage in nursing practice.

**Objective:**

This study examined the effects of cultural and other related factors on nurses’ intentions to use nursing information systems. The findings were expected to serve as an empirical base for further benchmarking and management of cross-strait nurses.

**Methods:**

This survey was conducted in two case hospitals (one in Taiwan and one in China). A total of 880 questionnaires were distributed (n=440 in each hospital).

**Results:**

The results showed effort expectancy had a significant effect on the intention to use nursing information systems of nurses in China (*P*=.003) but not nurses in Taiwan (*P*=.16).

**Conclusions:**

Findings suggest nursing managers should adopt different strategies to motivate cross-strait nurses to use nursing information systems. Promoting effort expectancy is more likely to motivate nurses in China than in Taiwan. This discrepancy is probably due to the less hierarchical and more feminine society in Taiwan.

## Introduction

### Research Background

Differences in political structures influence cultural development. Eveland et al [1] found cultural differences in political discussions and relationship strength. The natural divide between Taiwan and China, the Taiwan Strait, resulted in differences over time in their political systems and in culture and economy. Past studies on cross-cultural behavioral differences have primarily focused on the manufacturing sector [2-6]. Hofstede [7] reported that power distance was less salient in Taiwan than in mainland China. Power distance directly influences the perceived quality of medical services [8]. In other words, culture could play a key role in the perceived quality of cross-strait nursing services.

The shortage of nursing staff is a long-standing global problem. In Taiwan, this shortage is progressively becoming more serious because of Taiwan's rapidly aging population [9]. In China, the number of registered nurses surpassed 4 million at the end of 2018, but the workforce is still insufficient [10]. Recently, interactions between nursing staff on either side of the Taiwan Strait continue to increase. However, nursing training programs are different between Taiwan and China. Furthermore, both nurses in Taiwan and nurses in China are adopting nursing information systems which are designed according to what they have learned. Therefore, the cross-strait nursing management is becoming an important issue that needs to be properly addressed.

Adopting nursing information systems can strengthen the mechanisms that underlie patient safety, improve service efficiency, improve retention rate, reduce work load, and reduce management costs. Similar to other industries in which considerable investment in information systems is required to gain the competitive advantage, the medical industry also invests significant resources in information systems to maintain quality and improve performance. Nurses are responsible for inpatients’ physical and mental well-being, and they are likely to use nursing information systems frequently. Information competency is defined as the ability to find, evaluate, and use information efficiently and is important in electronic or paper-based systems. Information competency is essential for nurses to provide safer, more effective, and more efficient health care and it determines the success in implementing clinical information systems [11]. Research studies have found that nurse information competency can improve the efficient use of information systems and can enhance decision making about patient care [12]. Khezri et al [13] showed that nurses’ informatics competencies have a more critical impact on patient outcomes and organizational success than the information systems did, per se.

### Nursing Information Systems

Nursing information systems are designed to host all levels of data that nurses collect about nursing activities, resources, research, management, and education. The use of nursing information systems can help nurses provide (or acquire) accurate and real-time clinical information to (or from) patients, physicians, and other health care providers to ensure the provision of high-quality health care. A nursing information system is a computer-based system that collects, retrieves, stores, processes, displays, and communicates information in a timely manner to facilitate nursing care and resource management as well as providing information on patient health [14]. Once the nursing information system has been developed, several important issues emerge (eg, user acceptance, workflow standardization, and automatic operations of procedures). Nguyen et al [15] found that performance expectancy, effort expectancy, and social influence are positively correlated with intention to use nursing information systems, and nurses reported that the nursing information system could reduce their documentation time and enable them to spend more time with patients. Handayani et al [16] systematically reviewed 56 previous studies on user acceptance factors of hospital information systems and related technologies and identified perceived usefulness or performance expectancy, perceived ease of use or effort expectancy, system quality, and subjective norms or social influence (15 frequent factors) as factors.

### National Culture

A thorough understanding of the background, characteristics, and values of organizational members from different cultures can offer organizations new opportunities to gain competitive advantages and avoid potential threats in information systems. Hofstede et al [17] classified culture into different categories based on six dimensions, namely, power distance, uncertainty avoidance, individualism, masculinity, long-term orientation, and indulgence and the scores on these six dimensions were computed using formulas for 76 countries. Across the Taiwan strait, the same Chinese language is spoken. However, there are cultural differences between these two regions, therefore, cross-strait staff undergo substantial cross-cultural adaptations [18]. The scores that China and Taiwan obtained on the dimensions of power distance, masculinity, uncertainty avoidance, and indulgence revealed that there were substantial differences between the two cultures. Hofstede’s interpretations of the six dimensions are shown in Table 1.

In Table 1, scores between Taiwan and China are largely different in the dimensions of power distance, masculinity, uncertainty avoidance, and indulgence. Chen et al [19] later indicated that cultural differences of individualism, uncertainty avoidance, masculinity, and long-term orientation are more obvious between Taiwan and China. Lin et al [20] used masculinity, collectivism, and uncertainty avoidance to explore the influences on social media users in Taiwan acquiring and sharing health-related information.

In this study, the common dimensions of prior studies—masculinity and uncertainty avoidance—were chosen to verify the culture differences between the two groups of nursing information system users and were used as moderators to examine how their intention on using nursing information systems was influenced accordingly.

**Table 1:** Six-dimensional model of the national culture of Taiwan and China based on Hofstede [7].

Dimension	Taiwan (score)	China (score)
Power distance	People accept the established order without requiring further justification (58).	People believe that inequalities among people are acceptable (80).
Individualism	A collectivistic society in which long-term commitments are shared with close group “members” such as family members (17).	A collectivist culture in which people act in accordance with the interests of the group rather than personal interests (20).
Masculinity	A slightly feminine society in which the quality of life is a sign of success and standing out from the crowd is not desirable (45).	A patriarchal society that is driven by competition, achievement, and success, whereby people may prioritize work over family and leisure (66).
Uncertainty avoidance	People have a strong preference for the avoidance of uncertainty, resulting in an emotional need for rules (69).	A culture in which people are comfortable with ambiguity and believe that truth is relative (30).
Long-term orientation	People have a pragmatic long-term orientation and an ability to adapt traditions to modern contexts (93).	People believe that truth, to a great extent, depends on a given situation, context, and time (87).
Indulgence	No preference on this dimension (49).	People are restrained by social norms and believe that indulging themselves is somewhat wrong (24).

### Information Literacy

Information literacy is a set of competencies and skills that help individuals effectively locate, evaluate, and use the required information. Health care organizations obtain real-time information that can be used for efficient work processes, better patient safety, clinical decision making, and effective health care. Evidence-based nursing practice requires resilient, innovative, accurate, and helpful real-time information, not only from the hospital-wide record systems with quality and performance indicators but also from advanced nursing-related research evidence, and transfers that information to existing routine nursing practice pertaining to patient care. Past studies have found that, in their workplaces, nursing staff often lack the time, resources, and tools to make use of comprehensive real-time information to make decisions regarding patient care. Kleib et al [21] found that mean informatics competency scores were significantly related to the age, educational qualification, and years of work experience of nurses. Abdrbo [22] recommended the incorporation of information competencies into nursing programs and the introduction of a major course on nursing informatics. In summary, information literacy is important for nurses on their acceptance of the nursing information system.

The unified theory of acceptance and use of technology (UTAUT) framework is widely used to facilitate research on the adoption of an information system or information technology. UTAUT [23] was proposed and validated on electronic medical records system. Since both the systems and users of nursing information systems and electronic medical records systems are different, adjustments needed to be made to the UTAUT model to incorporate our research goal. In this study, our research model was developed based on the UTAUT by adopting the key variables of performance expectancy, effort expectancy, social influence, and intention to use. Meanwhile, two aforementioned constructs, culture and information literacy, were added to explore the nurses’ intention of using nursing information systems.

## Methods

### Participants

In this study, we selected two hospitals, Taichung Veterans General Hospital (in Taiwan) and Suzhou Tertiary Hospital (in China). Both hospitals have nursing information systems that are considerably more advanced than those of other hospitals in their respective countries. However, the nursing information systems vary considerably in their scopes. The nursing information systems of Taichung Veterans General Hospital have more functions and wider applications regarding safety and quality assurance. Both nursing information systems and their host hospitals are described.

Taichung Veterans General Hospital is a medical center in central Taiwan that has 500 beds and 3700 employees. It can serve 7000 outpatients and 190 patients/day at the emergency room. The development of its hospital information systems began in 1970, and nursing information systems were implemented in 1993. This was the first of its kind implemented in the nursing department in Taiwan. Subsequently, a wide range of information-based systems were implemented in the department, including a web-based nursing information systems, mobile nursing stations, computerized nursing shift guidelines, a computerized discharge plan, digital signatures on digital medical records, an emergency room nursing information systems, a nutritional evaluation system, a social welfare consultation services support system, a system for patient recognition during blood transfusion, a newborn care evaluation tool, and six types of pain assessments. In 2016, this hospital’s nursing information systems received the Safety and Quality Certificate Silver Award under the category of National Health and Medical Quality.

In 1988, Suzhou Tertiary Hospital was founded as the General Hospital for the Nuclear Industry and for Sino-French Friendship. It has 1976 employees, including 1653 health professionals, 18 teaching doctors, and 105 teaching postgraduates. The initial information systems implemented in this hospital were electronic health care record systems, which consisted of computerized nursing forms, vital-sign records, and a credit management system. The first version of the nursing management information system was also implemented. Other supporting systems have also been implemented such as statistical analysis of adverse events, label printing, specimen collection and confirmation information systems, supply center information systems, the quality of health and medical records, blood transfusion system, drug tracking system, nursing quality management system, emergency information system, and catheter room information system.

Ethical approval to conduct this study was approved by the Institutional Review Board of Taichung Veterans General Hospital (serial number: CE16137A#1). In accordance with the Institutional Review Board regulations of this study, the data are not publicly available.

Nursing staff members who had at least two years of work experience in the two aforementioned hospitals and prior experience with the nursing information systems were invited for study. All the selected nursing members were informed that their participation was voluntary, that they had the right to withdraw from the study at any time, and that their personal information remained confidential.

### Study Design

**Figure 1:**
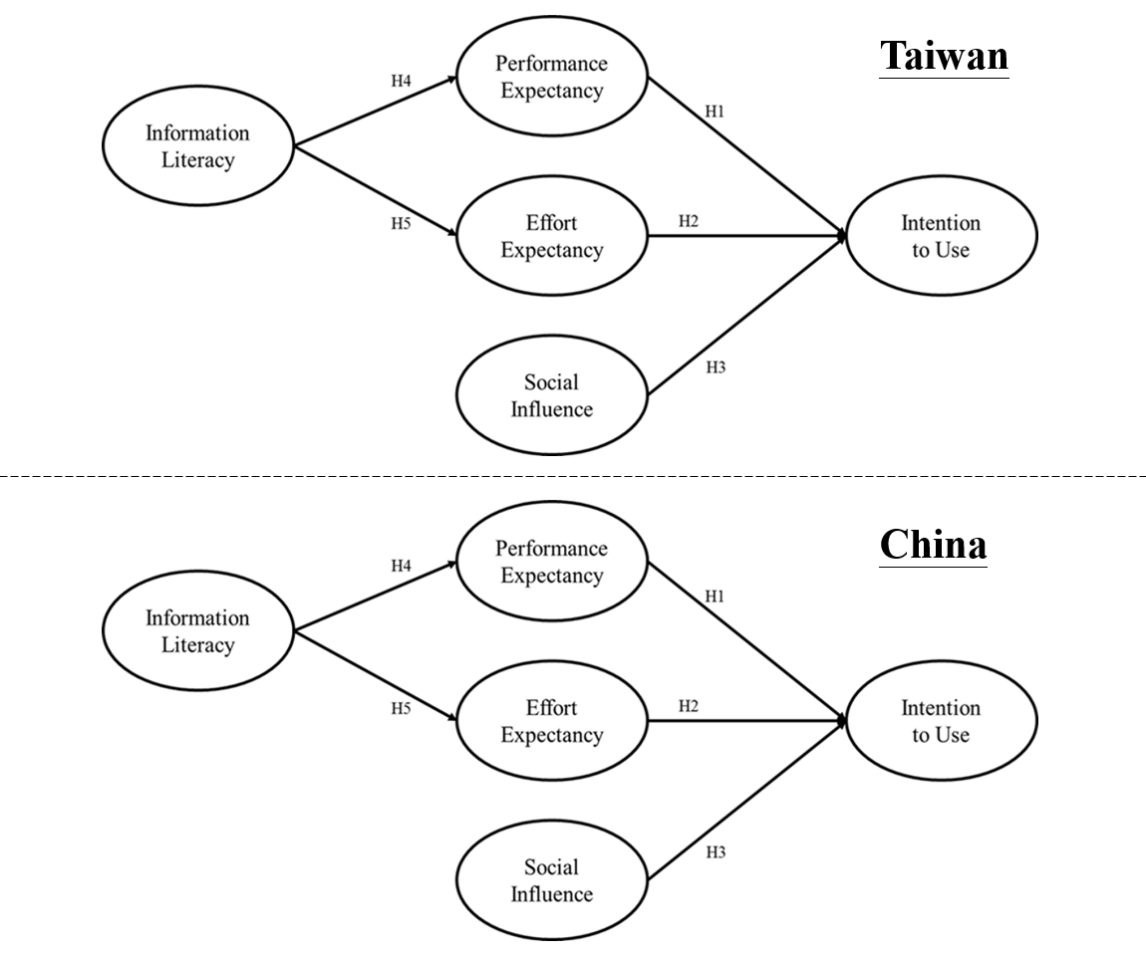
Research framework.

Nursing information systems in the two case hospitals were not identical. The purpose of the study was to investigate system usability instead of the systems per se, hence we adopted the key constructs of the UTAUT related to system usability. Since both hospitals had a sufficient number of up-to-date facilities for the functioning of nursing information systems, we examined only 3 variables that influence users' intention of the UTAUT (ie, performance expectancy, effort expectancy, and social influence), and in addition, used information literacy and culture variables to ascertain the acceptance and usage of nursing information systems. Performance expectancy was defined as the extent to which an individual believes that using nursing information systems will help enhance job performance. Effort expectancy was defined as the ease-of-use expectancy in using nursing information systems. Social influence was defined as the extent to which an individual believes that others expect them to use the nursing information systems. The research model is shown in Figure 1. The research model was tested differently between the Taiwan and China groups; therefore, culture was used as moderator to examine all the relations of the research framework. Based on the research model shown in Figure 1, we formulated the following hypotheses.

Hypothesis 1: Nurses who believe nursing information systems will enhance their performance are more willing to use the system.

Hypothesis 2: Nurses who expected a high degree of ease in using the nursing information systems will be more willing to use the systems.

Hypothesis 3: Nurses who perceive other caregivers who use nursing information systems receiving higher levels of social influence are more willing to use the system.

Hypothesis 4: Nurses with higher levels of information literacy will have higher performance expectancies of nursing information systems.

Hypothesis 5: Nurses with higher levels of information literacy will expect higher degrees of ease in using nursing information systems.

The model was validated by data obtained separately by the two groups of nurses. Culture is a moderator that moderates the five relationships among variables of the research model. As previously mentioned, the common dimensions used in prior studies, ie, masculinity and uncertainty avoidance, were chosen to verify the cultural differences of the two groups of nursing information system users. A two-sample *t* test showed that masculinity (*t*_797_=–5.15, *P*=.002) and uncertainty avoidance (*t*_797_=4.45, *P*=.02) dimensions were significantly different between two groups (Multimedia Appendix 1). Therefore, the masculinity and uncertainty avoidance dimensions were used as moderators in this study.

### Instrument Development

Based on the literature review, we first developed a preliminary list of measurement items. Subsequently, we modified the item contents to enhance the reliability and validity of the indicators. Participants were instructed to respond to each item on a 5-point Likert scale (with 1 = strongly disagree, 5 = strongly agree). In order to examine the validity of the questionnaire, 3 experts (2 from China and 1 from Taiwan) with substantial experience in the field of nursing information systems were invited. In addition, 2 academic experts who taught and published papers on nursing information systems developments were invited to form an expert panel. They were asked to examine the research framework, format of the measurement items, length of the instrument, and wording of the scale items. Based on the feedback and suggestions that were provided by the 5 experts, the questionnaire was modified to enhance the validity of the scale items. The modified measurement items were pretested on 3 nurses. Next, a pilot study was conducted and 15 experienced nursing information systems users in each of the two hospitals were invited to participate in the pilot study. According to Gorsuch [24], the number of participants should be at least 5 to 10 times the number of items, and the sample size should be greater than 100 whenever factors analyses are performed on the acquired data. The questionnaire consisted of 34 items related to the research model (Multimedia Appendix 1) and 6 items of demographic information including age, educational level, job title, work experience, and level of computer skills. Finally, we distributed online or paper questionnaires (N=880; Taiwan: n=440; China: n=440). SPSS statistical software (version 22.0; IBM Corp) and SmartPLS (version 3.2.6; SmartPLS GmbH) [25] were used to conduct statistical analyses. In this study, the bootstrapping method with 5000 resamples was used to determine the level of statistical significance of each hypothesis [26].

### Reliability and Validity

We employed the partial least squares approach of structural equation modeling to examine the psychometric properties of the assessment and path coefficients of the structural equation. This analysis was executed using SmartPLS. Reliability analysis was conducted by computing Cronbach α and composite reliability. Scales were considered to have satisfactory reliability if the Cronbach α and composite reliability of each construct were higher than 0.8 and 0.70 [26,27], respectively. In this study, the Cronbach α and composite reliability of all the constructs exceeded 0.8. Therefore, the scales had a satisfactory reliability. Furthermore, the average variance extracted value of each construct should be greater than 0.50, the variance caused by measurement error, to have convergent validity [26]. In this study, the average variance extracted of all the constructs exceeded 0.64. Therefore, the scales had a reasonable convergent validity. Finally, the values of discriminant validity, the square root of the average variance extracted value of each construct in the model, should all be greater than the estimated correlations between the respective construct and other constructs. The square root of the average variance extracted value of each construct is shown in the Multimedia Appendix 1. Items with loadings less than 0.4 were excluded from the final analysis. The results of reliability and validity analyses are shown in Multimedia Appendix 1. The instrument demonstrated acceptable composite reliability and convergent and discriminant validity.

## Results

### Overview

Questionnaires with invalid responses were discarded prior to statistical analyses. Valid responses were obtained from a total of 799 participants (Taiwan: n=400, China: n=399).

### Descriptive Statistics

Table 2 shows the demographic characteristics of the participants, including their age, educational level, job title, work experience, and level of computer skills.

Most nurses in our study were women (394/400, 98.5%) with an intermediate level of computer literacy (299/400, 74.8%). Nurses from the hospital in Taiwan were older (older than 30 years: 264/400, 66.0%), had a job title of nurse (305/400, 76.3%), had a bachelor’s degree or higher educational qualification (358/400, 89.6%), and had longer work experience (216/400 or 54.1% had more than 10 years of work experience). In contrast, most of the nurses from the hospital in China were younger (older than 30 years: 179/399, 44.8%), had a job title of team leader (242/399, 60.7%), had a bachelor’s degree or higher educational qualification (274/399, 68.7%), and had shorter work experience (>10 years: 142/399, 35.5%).

The high ratio of nurses with bachelors’ degrees or higher, 89.6% (358/400) for Taiwan and 68.7% (274/399) for China, may explain their intermediate level of computer literacy, because courses of introduction to computer software or basic programming are taught as compulsory general courses at college level on both sides.

**Table 2:** Demographic characteristics of participants from Taiwan and China.

Characteristic, category		Taiwan (n=400), n (%)	China (n=399), n (%)
**Gender**			
	Male	6 (1.5)	6 (1.5)
	Female	394 (98.5)	393 (98.5)
**Age (years)**			
	<25	20 (5.0)	45 (11.3)
	26-30	116 (29.0)	175 (43.9)
	31-35	87 (21.8)	94 (23.6)
	36-40	44 (11.0)	42 (10.5)
	41-45	58 (14.5)	27 (6.8)
	46-50	45 (11.3)	9 (2.3)
	>50	30 (7.5)	7 (1.8)
**Educational level**			
	High school	0 (0.0)	3 (0.8)
	Community college	42 (10.5)	122 (30.6)
	University bachelor’s degree	303 (75.8)	268 (67.2)
	Master’s degree or higher	55 (13.8)	6 (1.5)
**Job title**			
	Nurse	305 (76.3)	47 (11.8)
	Team leader	6 (1.5)	242 (60.7)
	Vice head nurse	18 (4.5)	94 (23.6)
	Head nurse or higher	13 (3.3)	16 (4.0)
	Other	58 (14.5)	0 (0.0)
**Work experience (years)**			
	1-2	0 (0.0)	11 (2.8)
	2-5	72 (18.0)	77 (19.3)
	5-10	112 (28.0)	169 (42.4)
	10-15	67 (16.8)	66 (16.5)
	>15	149 (37.3)	76 (19.0)
**Level of computer skills**			
	High	57 (14.2)	33 (8.3)
	Intermediate	299 (74.8)	305 (76.4)
	Low	44 (11.0)	61 (15.3)

### Structural Model Analysis

**Figure 2:**
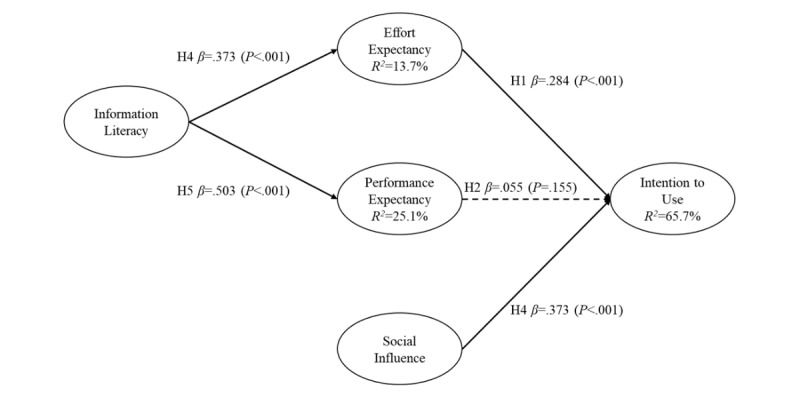
Path analysis results of Taiwan.

**Figure 3:**
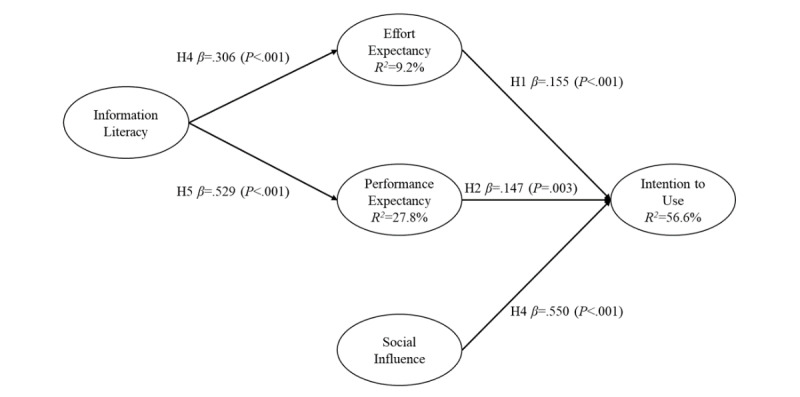
Path analysis results of China.

Figure 2 and Figure 3 show the estimated path coefficients and explanatory R^2^ values separately for the samples from the hospitals in Taiwan and China.

Results from analyses of the sample of nurses from the hospital in Taiwan supported all our hypotheses, except hypothesis 2 (β=0.055, *t*=1.5, *P*=.16). Performance expectancy (β=0.284, *t*=5.6, *P*<.001) and social influence (β=0.533, t=10.0, *P*<.001) had a positive effect on nursing information systems usage intentions. Hypothesis 1 and hypothesis 3 were hence supported. However, effort expectancy was not significantly related to nursing information systems usage intentions. Hypothesis 2 was hence not supported. Information literacy had a positive effect on both performance (β=0.373, *t*=5.1, *P*<.001) and effort expectancy (β=0.503, *t*=8.9, *P*<.001). Hypothesis 4 and hypothesis 5 were hence supported. Overall, the model explained 65.7% (R^2^=0.657) of the variance in nursing information systems usage intentions.

Results from analyses of the sample from the hospital in China supported all our hypotheses. Performance (β=0.155, *t*=3.5, *P*<.001), effort expectancy (β=0.147, *t*=2.9, *P*=.003), and social influence (β=0.550, *t*=11.1, *P*<.001) had a positive influence on nursing information systems usage intentions. Hypothesis 1, hypothesis 2, and hypothesis 3 were hence supported. Information literacy had a positive influence on both performance (β=0.306, *t*=3.3, *P*<.001) and effort expectancy (β=0.529, *t*=8.8, *P*<.001). Hypothesis 4 and hypothesis 5 were hence supported. Overall, the model explained 56.6% (R^2^=0.566) of all the variance in nursing information system usage intentions. The comparison of the results of path analyses and hypothesis testing between the samples are shown in Table 3.

**Table 3:** Comparison of hypotheses test results.

Hypothesis	Nurses in Taiwan				Nurses in China				
	β	*t* value	*P* value	Decision		β	*t* value	*P* value	Decision
1. Nurses who believe the NIS^a^ will enhance their performance are more willing to use the system.	0.284	5.557	<.001	Support		0.155	3.535	<.001	Support
2. Nurses who expect a high degree of ease in using the NIS will be more willing to use the system.	0.055	1.461	.16	Reject		0.147	2.959	.003	Support
3. Nurses who perceive other caregivers who use the NIS have a higher level of social influence than them are more willing to use the system.	0.533	10.021	<.001	Support		0.550	11.135	<.001	Support
4. Nurses with higher levels of information literacy will have higher performance expectancies of the NIS.	0.373	5.083	<.001	Support		0.306	3.276	<.001	Support
5. Nurses with higher levels of information literacy will expect higher degree of ease in using the NIS.	0.503	8.946	<.001	Support		0.529	8.828	<.001	Support

^a^NIS: nursing information system

All the relationships in the research framework between the groups were similar except the effect of effort expectancy on nursing information systems usage intentions was stronger with participants from the hospital in China (β=0.147, *t*=3.0, *P*=.003) than with participants from the hospital in Taiwan (β=0.055, *t*=1.5, *P*=.16). With regard to cultural effects, this indicated that, assuming all else equal, the participants from the hospital in China were more prepared to take risks (lower uncertainty avoidance) and more competitive (greater masculinity) than the participants from the hospital in Taiwan and hence showed greater ease to use a nursing information system having a significant effect on their intention to use nursing information system. In contrast, nurses from the hospital in Taiwan lived in a more risk averse, less competitive, and slightly feminine society. They would likely have used nursing information systems regardless of the degree of ease in using the system. Furthermore, nurses from the hospital in Taiwan may have more realistic expectations of the amount of effort required in using the nursing information systems.

## Discussion

### Culture Influences Nursing Information Systems Usage Intention

In this study, the key constructs of the UTAUT, cultural differences, and information literacy were integrated to develop a better theoretical framework through which factors affecting nursing information systems usage intentions can be examined. First, the results confirmed Nguyen et al’s [15] findings using the UTAUT model. Furthermore, this study is the first of its kind in exploring cultural influences on nursing information systems usage intentions. Results revealed that performance expectancy and social influence were significant predictors of nursing information system usage intentions. In other words, nurses are likely to use a particular system if they believe that it will enhance their performance and fulfill social expectations. In addition to promoting performance expectancies, nursing managers should capitalize on the social influences from peer groups and colleagues to promote usage of nursing information systems among both nurses in Taiwan and nurses in China. It is also important for managers to be aware that the positive effects of performance expectancy on the motivation to use nursing information systems are stronger among nurses in Taiwan than among nurses in China.

However, operational ease has an effect only on users who belong to societies (eg, China) that are more accepting of inequality and people are more driven by competition. Therefore, hospital managers should promote effort expectancies to motivate nurses in China to use nursing information systems. For example, helping nurses complete their tasks more easily and improving the quality of their work should be regarded as the most important objective in nursing information system designs. Samples of nurses in Taiwan and in China showed that information literacy had a positive effect on performance and effort expectancies. This indicates that stronger information literacy promotes expectancies that the use of nursing information systems will improve performance and not be effortful. This in turn likely enhances nursing information systems usage intentions.

### Research Limitations

This study collected data via both online and paper questionnaires. Since the questionnaire was self-reported by respondents, it is hard to confirm the authenticity of respondents' answers. In addition, assuming everything else being equal, culture is expected to be the main reason why effort expectancy was related to nursing information system use only in China but not in Taiwan. Since the study was conducted in an open environment instead of in an experimental laboratory, applying the results of this study may need caution.

### Direction of Future Studies

Cross-strait nurse movement is mainly from mainland China to Taiwan, due to Taiwan’s nurse shortage. However, this study also found a shortage of nurses in mainland China (nursing managers and leaders) which led to nurse movement from Taiwan to mainland China. In addition, both Chinese and Taiwanese medical systems are hybrid models and each hospital may develop its own medical information systems. Therefore, not only cross-strait but also cross-hospital nurses need to adopt new nursing information systems. This study explored the impact of culture and UTAUT key variables on nurses’ nursing information systems usage intentions to develop strategies and refine nursing management and care processes. Future studies can explore more factors affecting perceived ease of use and satisfaction with using nursing information system, to incorporate motivation theory, and to extend the present study findings to further promote the acceptance of nursing information systems among users and mitigate the nursing workforce shortage.

### Conclusions

Managing an international workforce is a global issue and understanding cultural differences can help managers run their organizations smoothly and efficiently. In this regard, this study has two key contributions. First, we explored perceptions of and knowledge about nursing information systems of nurses in China and nurses in Taiwan. With knowledge of the differences between nurses on both sides of the Taiwan Strait, hospitals can be in a position to better encourage nurses to use nursing information systems more efficiently while providing care. Meanwhile, differences in the scope of the nursing information system between both countries can provide focus points for cross-strait on the job nurse training programs. The consequence is likely to further enhance the quality of services when hiring cross-strait nurses.

Additionally, cross-strait system vendors can make use of our findings to better understand behavioral differences in the acceptance and use of the nursing information systems between nurses in Taiwan and nurses in China. System vendors should modify their system development strategies to fit the cross-strait nursing industry better. For example, more intuitive user interfaces such as drop-down menus and popup help desks or windows are suggested to increase the degree of ease in using nursing information systems. Finally, this study is one of the first attempts to explore cultural influences on the cross-strait nursing industry. Cultural differences are often discussed within the context of the manufacturing industry, but only Li et al [28] have explored such differences in the behavioral intentions of health care employees. In this regard, this study expands the scope of this domain of research and contributes new findings to the existing literature.

## Appendix

Multimedia Appendix 1 Supporting information.

## Abbreviations

UTAUT: unified theory of acceptance and use of technology
