# Supplementary material for: Cultural Impact on the Intention to Use Nursing Information Systems of Nurses in Taiwan and China: Survey and Analysis
Source: J Med Internet Res. 2020 Aug 12;22(8):e18078. doi: 10.2196/18078 (PMC7450378; doi:10.2196/18078)
Supplement: Multimedia Appendix 1 [file jmir_v22i8e18078_app1.pdf]

Multimedia Appendix 1.

Supporting Information

S1 Table. Two-sample t-test of culture dimension.

**Culture Dimension**	***T* value *(df)***	***P* value**
**Uncertainty Avoidance**	4.450 (797)	0.020*
**Masculinity**	-5.151 (797)	0.002*

Note: **P*< 0.05

S2 Table. Items of the Questionnaire.

**Information literacy (Kiliç-Çakmak, 2010)**
I can define the information that I need.
I can limit search strategies based on the subject, language, and date.
I can decide where and how to find the information that I need.
I can use internet search tools (e.g., search engines, directories)
I can determine the authoritativeness, correctness, and reliability of information sources.
I can synthesize newly gathered and previously retrieved information.
I can determine the content and form the parts (introduction, conclusion) of a presentation (written, oral).
I can evaluate the quality of information-seeking processes and resultant products.
**Uncertainty Avoidance and Masculinity (Wu & Zhang, 2013)**
U1: How often do you feel nervous or tense?
U2: Do you agree that a good manager without having a precise answer to every question that a subordinate may raise about his or her work?
U3: Do you agree that competition between employees usually does more good than harm?
U4: Do you agree that organizational rules should not be broken, even if one is acting in the best interests of the organization?
M1: How important is it to you to work with pleasant people?
M2: How important is it to you to have opportunities for promotion?
M3: How important is it to you to be recognized for good performance?
M4: Do you agree that, when people fail in life, it is often their own fault?
**Performance expectancy (Simeonova, Bogolyubov, Blagov, & Kharabsheh, 2014)**
The nurse information system will be useful to my work.

The nurse information system helps me complete my tasks more quickly.
The nurse information system increases my productivity.
If I use the nurse information system, my chances of successfully completing a task will increase.
**Effort expectancy (Simeonova et al., 2014)**
My interaction with the nurse information system will be clear and understandable.
It will be easy for me to become a skilled user of the nurse information system.
The nurse information system will be easy to use.
I will find it easy to learn to operate a nurse information system.
**Social influence (Simeonova et al., 2014)**
What do those in your social surroundings think about the nurse information system?
People who influence my behavior think that I should use the nurse information system
I use the nurse information system because most of my colleagues do.
The managers are supportive of the use of the nurse information system.
In general, the hospital supports the use of the nurse information system.
**Intention to use (Tarhini, Hone, & Liu, 2013)**
I will use the nurse information system when it becomes available to me.
I intend to use the nurse information system when it becomes available to me.
If I have access to a nurse information system, I will use it.
I expect to use a nurse information system soon.
In general, I plan to frequently use a nurse information system as a part of my work.

S3 Table. Correlation Matrix for Discriminant Validity based on the Results of Partial Least Squares Structural Equation Modeling.

**Taiwan Group**					
**D**	**IL**	**IU**	**EE**	**PE**	**SI**
**IL**	**0.822**				
**IU**	0.400	**0.882**			
**EE**	0.503	0.667	**0.871**		
**PE**	0.374	0.718	0.704	**0.871**	
**SI**	0.434	0.780	0.758	0.726	**0.761**
**China Group:**					
**D**	**IL**	**IU**	**EE**	**PE**	**SI**
**IL**	**0.806**				
**IU**	0.478	**0.842**			
**EE**	0.529	0.600	**0.848**		
**PE**	0.306	0.512	0.491	**0.847**	
**SI**	0.551	0.731	0.686	0.520	**0.756**

Note: D, dimension; IL, information literacy; IU, intention to use; EE, effort expectancy; PE, performance expectancy; SI, social influence. Values presented along the diagonal are the square roots of average variance extracted values, and the values below the diagonal are correlation coefficients between the factors.

S4 Table. Results of Reliability and Validity Analyses using Partial Least Squares Structural Equation Modeling.

Dimension	Item	Taiwan Group				China Group			
		FL	AVE	CR	CA	FL	AVE	CR	CA
**Information Literacy**	IL_1	0.751	0.676	0.943	0.931	0.770	0.649	0.936	0.922
	IL_2	0.822				0.822			
	IL_3	0.836				0.857			
	IL_4	0.785				0.803			
	IL_5	0.850				0.673			
	IL_6	0.891				0.869			
	IL_7	0.820				0.815			
	IL_8	0.836				0.822			
**Performance Expectancy**	PE_1	0.845	0.759	0.940	0.921	0.796	0.717	0.927	0.901
	PE_2	0.879				0.851			
	PE_3	0.859				0.894			
	PE_4	0.888				0.874			
	PE_5	0.885				0.814			
**Effort Expectancy**	EE_2	0.862	0.759	0.926	0.894	0.791	0.719	0.911	0.869
	EE_3	0.822				0.828			
	EE_4	0.907				0.901			
	EE_5	0.891				0.867			
**Social Influence**	SI_1	0.776	0.580	0.872	0.817	0.722	0.572	0.869	0.812
	SI_2	0.765				0.769			
	SI_3	0.570				0.624			
	SI_4	0.841				0.847			
	SI_5	0.824				0.800			
**Intention to Use**	IU_1	0.884	0.777	0.946	0.928	0.833	0.710	0.924	0.898
	IU_2	0.888				0.863			
	IU_3	0.892				0.867			
	IU_4	0.879				0.807			
	IU_5	0.865				0.842			
